# Screening is associated with lower mastectomy rates in eastern Switzerland beyond stage effects

**DOI:** 10.1186/s12885-021-07917-2

**Published:** 2021-03-06

**Authors:** Christian Herrmann, Rudolf Morant, Esther Walser, Mohsen Mousavi, Beat Thürlimann

**Affiliations:** 1 Cancer Registry of Eastern Switzerland, Cancer League of Eastern Switzerland, St. Gallen, Switzerland; 2 Cancer League of Eastern Switzerland, St. Gallen, Switzerland; 3 Division Oncology-Haematology, Department of Internal Medicine, St. Gallen, Switzerland; 4 Breast Centre St.Gallen, Cantonal Hospital, St. Gallen, Switzerland

**Keywords:** Mammography screening programme, Public health, Mastectomy, Epidemiology, Breast cancer, Switzerland

## Abstract

**Background:**

A recent study found an influence of organized mammography screening programmes (MSPs) on geographical and temporal variation of mastectomy rates. We aimed to quantify the effect on the example of one of the cantonal programmes in Switzerland.

**Methods:**

We used incidence data for the years 2010–2017 from the cancer registry of Eastern Switzerland. We included women with invasive-non-metastatic breast cancer (BC) in the screening age group 50–69-year-olds in the canton of St.Gallen. We compared mastectomy rates among cancer patients detected through the organised screening programme (MSP) vs. otherwise detected by stage.

**Results:**

MSP-detected patients in St.Gallen presented with lower stages. 95% of MSP-detected had stages I-II vs 76% of Non-MSP-detected. Within all non-metastatic stage, tumour size and nodal status groups, MSP-detected patients had lower mastectomy rates, overall 10% vs 24% in 50–69-year-old non-participants. Their odds of receiving a mastectomy are about half of the Non-MSP-detected (OR = 0.48, *p* = 0.002).

**Conclusions:**

Our study showed that MSPs have a positive effect on lowering mastectomy rates. Screening participants are significantly less likely to receive a mastectomy compared to non-participants, which must be attributed to additional factors than just lower stages. Lower mastectomy rates lead to a higher quality of life for many patients.

## Background

Female breast cancer (BC) is the most frequent cancer of females in Switzerland as in most European countries [3]. Switzerland is a small confederation of 26 relatively autonomous states called cantons. Most health care policies are developed at the cantonal level and there is a large geographical variation in health expenditures, reflecting disparities in resource utilisation. The decision to initiate an organised mammography-screening programme had been taken by the St.Gallen parliament in 2008.

The traditional type of breast surgery has been mastectomy, until results from well-designed randomized trials in the 1980s showed, that less mutilating surgical procedures incorporating radiotherapy had similar rates of overall survival and disease-free survival compared to mastectomy. Preserving the most part of the breast (breast-conserving surgery) like lumpectomy or quadrantectomy was aimed at optimal disease control while preserving the quality of life [22]. A study-update with a 20-year follow-up confirmed the preliminary findings, establishing the concept of breast conservation as a standard of care [21].

Only few publications about recent mastectomy trends exist for Europe. Due to widespread use of screening, trends in absence of screening are difficult to determine. In Norway a general downward trend in mastectomy rates was described for all age groups (40–49,50-69,70–79) [20] The authors describe an increase in breast surgery and mastectomy rates with the start of population based screening. In contrast, in a recent study [11], we showed that mastectomy rates declined for patients in Switzerland aged 50–69 and 70+ and remained stable for those under 50, all with important geographical differences. Mastectomy rates in the French language region were observed to be significantly lower; this is the language region where mammography screening programmes started the earliest. However, when including the existence of population-based mammography screening programmes in our model, we showed an additional significantly reduced rate of mastectomies of about 13%. Population-based mammography screening programmes (MSPs) started at very different time points in Switzerland. The first pilot programme was established in 1993 and by 2001 only three cantons had established screening programmes. Until 2012 10 cantons had screening programmes for more than 10 years and 3 for at least 5 years. It has been shown, that screening programmes lead to a downshift in stage distribution in the respective cantons [4, 5]. And Ess et al. showed that breast cancer patients with lower stages had a significantly lower rate of mastectomies in Switzerland [6].

We aimed to investigate, whether the lower rate of mastectomies for cantons with existing mammography screening programmes is due to the stage shift.

## Methods

We used incidence data for the years 2010–2017 from the cancer registry of Eastern Switzerland. The dataset included information on age, the reason for cancer detection (screening programme (MSP) vs. otherwise (Non-MSP)), diagnosis and TNM-stage information. The cancer registry provided also data on surgical treatments. Mastectomy was also assigned if it was the final type of surgery within primary treatment, e.g. after several breast conserving surgeries without disease free margins. The data from the registry are considered to be highly complete [13]. When information is missing, the registry will follow-up with the treating physicians in order to complete the registration. Only for 0.3% of breast cancer cases in the past two decades no information can be retrieved, so called death-certificate-only (DCO) cases.

We included women with invasive BC in the screening age group 50–69-year-olds in the canton of St.Gallen. We excluded patients with unknown stage information (*n* = 9, 0.7%). We compared mastectomy rates by stage for non-metastatic disease among patients whose tumours were detected by the organised screening programme (MSP) of St.Gallen and patients not detected through the MSP. The second group comprises of all women in the screening age group who had a diagnosis of cancer and may have participated in the screeing programme, but whose cancer was detected outside of the MSP. In the latter analysis, we also excluded patients with non-curative treatment intentions (*n* = 16, 1.2%). We confirmed that age was not a confounder in our analysis. We calculated the age-stratified Mantel-Haenszel combined odds ratios (OR) and assessed homogeneity of stratum odds with Χ^2^ tests. This result was also confirmed by logistic regression where age did not significantly improve model fit and showed a *p*-value of 0.28 for its OR of 1.02. Therefore we used an unconditional logistic regression not including age to assess the ORs.

We calculated the weighted average mastectomy ratio of MSP-detected over Non-MSP-detected rates to estimate the mastectomy rate among MSP-detected patients if the stages were distributed the same as in the Non-MSP group. As statistical tests, we used Χ^2^ and Fisher’s exact test where frequencies were below 5.

There was no primary data collection during this project. In this project anonymised and routinely collected data is used, collected as part of a cantonal cancer registration program, and aggregated prior to analysis. Therefore, according to federal regulations, this data can be used in epidemiological studies without additional ethics committee approval.

## Results

**Fig. 1:**
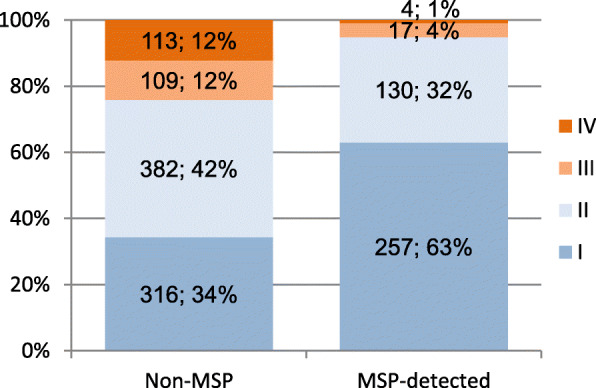
Stage distribution among BC patients by detection status in St.Gallen, in total numbers and percent

There were 1328 female breast cancer patients aged 50–69 years in the canton of St.Gallen in 2010–2017. 408 (31%) were detected due to the MSP. Stages in MSP-detected patients were lower than in Non-MSP-participants (*p* < 0.001, Fig. 1).

**Fig. 2:**
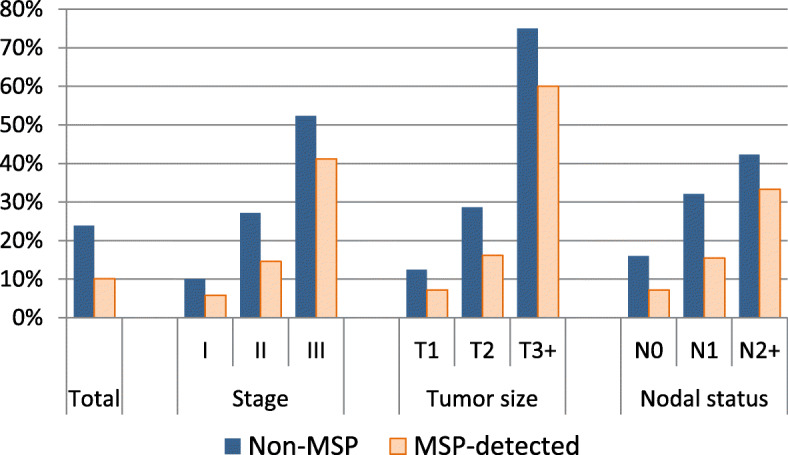
Mastectomy rates by stage and detection status

One thousand one hundred ninety-five patients had no metastasis present at diagnosis and were treated with curative intent, 404 (34%) of which were MSP-detected. While only 10% of MSP-detected patients received a mastectomy, 24% of patients with BC detected outside of the MSP did receive one. Also when stratifying by stage, tumour size or nodal status, MSP-detected patients had lower mastectomy rates throughout. (Table 1, Fig. 2) Not all differences in mastectomy rates were significant when considering Bonferroni corrections for multiple testing. We displayed all *p*-values in Table 1. Especially those categories where rates in the MSP group were based on 7 or fewer mastectomies (stage III, T3+ and N2+) had high *p*-values.

**Table 1:** Distribution and mastectomy rates of non-metastatic 50–69-year-old patients in the canton of St.Gallen 2012–2017 according to stage

Distribution of patients			Mastectomy rates			
stage	Non-MSP	MSP-detected	stage	Non-MSP	MSP-detected	*p*-value of difference
I	39%	64%	I	10%	6%	0.068
II	47%	32%	II	27%	15%	0.004
III	14%	4%	III	52%	41%^a^	0.390
***Total no.***	***807***	***404***	***total***	***24%***	***10%***	*< 0.001*
		*p* < 0.001				
T1	50%	75%	T1	12%	7%	0.024
T2	42%	23%	T2	29%	16%	0.015
T3+	7%	1%	T3+	75%	60%^a^	0.470
		*p* < 0.001				
N0	57%	72%	N0	16%	7%	< 0.001
N1	33%	24%	N1	32%	15%	< 0.001
N2+	10%	4%	N2+	42%	33%^a^	0.519
		*P* = 0.004				

MSP: Organized Mammography screening programme

Non-MSP: Patients invited to, but cancer not detected through MSP

MSP-detected: Patients where cancer was detected through MSP

^a^ rate based on less than 10 patients / less than 7 mastectomies

We calculated a weighted average mastectomy rate of 14% from stage-specific mastectomy rates of MSP-detected patients with the stage distribution of Non-MSP-detected patients as weights. So, if the stages in MSP-detected patients were distributed the same as in the Non-MSP group, calculated overall mastectomy rate among MSP-detected patients was with 14% still considerably lower than the 24% in the Non-MSP patients.

Table 2 shows the results of the logistic regression. Best model fit had the model including tumour size and mode of detection. In this model, the odds of receiving a mastectomy are less than half in MSP-detected patients (OR = 0.48, *p* = 0.002). Therefore, only a part of the total difference in mastectomy rates can be attributed to the stage difference.

**Table 2:** Logistic regression results of mastectomy rates of non-metastatic 50–69-year-old patients in the canton of St.Gallen 2012–2017 by stage and detection type

		Odds ratio	*p*-value	Model AIC
**stage:**	I	(reference)		654.4
	II	3.10	< 0.001	
	III	8.20	< 0.001	
**detection type:**	by MSP	0.45	0.001	
				**636.9**
**tumour size:**	T1	(reference)		
	T2	2.70	< 0.001	
	T3+	19.01	< 0.001	
**detection type:**	by MSP	0.48	0.002	
				674.6
**nodal status:**	N0	(reference)		
	N1	3.06	< 0.001	
	N2+	3.69	< 0.001	
**detection type:**	by MSP	0.38	< 0.001	
				709.8
**detection type:**	Not by MSP	(reference)		
	by MSP	0.34	< 0.001	

MSP: Organized Mammography screening programme

Non-MSP: Patients invited to, but cancer not detected through MSP

MSP-detected: Patients where cancer was detected through MSP

## Discussion

The effect of lower mastectomy rates in screen-detected patients goes beyond the lower stage distribution in MSP-detected cancers. Patients with cancers detected through the MSP received consistently less likely a mastectomy and have overall half the odds as those not detected through the MSP.

Breast-conserving surgery (BCS) can lead to a higher quality of life for many patients compared to mastectomy. Mastectomy patients usually reported a lower body image and sexual functioning [16]. Compared with BCS, mastectomy is a more invasive procedure that sometimes results in complications such as infection, poor healing, and lymphedema and requires longer hospital stays [1]. BCS results in less discomfort and pain, but requires (time-consuming) radiation and surveillance by mammography and might result in higher anxiety about recurrence.

There are, however, several reasons of personal, medical or preventive nature to choose a mastectomy in contrast to a BCS. These reasons include an increased risk of being diagnosed with second cancer due to BRCA mutations, larger tumours, multiple areas of the breast affected by cancer, and inflammatory breast cancer. An imbalance of these factors among the two groups may contribute to the observed difference in mastectomy rates.

Also, BCS should, in most cases, be combined with radiotherapy to result in equivalent survival as mastectomies [8, 15]. Therefore, Mastectomies might also be chosen when radiation therapy is medically contraindicated, frequently after previous BCS with radiation therapy, or on a personal level, if the patient prefers to avoid radiotherapy, e.g. because of living far from facilities offering radiation therapy [14].

Patients with previous breast cancer are not permitted into the screening but are more likely to receive a mastectomy. However, the incidence of second breast cancer in Eastern Switzerland is low with 4.5% [23, 24] and can only explain part of the difference. As for distance to radiation therapy units, using urbanisation level as a proxy did not significantly influence mastectomy rates in Switzerland, however, using the surgeon and gynaecologist density did so [11]. An imbalance of these factors may have contributed to the difference.

Furthermore, the mammography screening programme in St.Gallen follows strict quality assurance guidelines and may preferentially refer patients to specialized breast centres. For women with early BC it has been shown, that surgeons with higher caseloads and in multidisciplinary settings, such as in breast centres, are associated with decreased mastectomy rates [10]. Specialized breast centres in Switzerland are certified and monitored by EUSOMA, a non-profit society that promotes evidence-based high quality care for breast cancer patients by multidisciplinary breast teams [2, 7].

Only for France, there were recently reported differences in mastectomy rates by type of detection and stage at the same tim e[12]. However, the authors had no TNM-information available and could not further differentiate stages in the same way as we did. They found very similar mastectomy rates, of 14% in local breast cancers (T*N0M0) within MSP and 24% in non-MSP detected patients. For node-positive breast cancer (T*N + M0) these rates were 32% in MSP and 45% in non-MSP detected patents respectively. Other studies established a time correlation of screening start and increase in breast surgery and mastectomies, such as in Norway [20] and Germany [19]. But these data lack a differentiation by stage and may very well be a consequence of different stage profiles or general stage shift in the population. In a Cochrane review from 2013 [9] the authors reiterated their finding from an earlier review [17], that screening increases the number of mastectomies by 20%. It has to be noted, that the meta-analysis of this findings is based on papers published from 1972 to 1999. Treatment guidelines have been constantly updated in the 20+ years that followed. Also, in rebuttal of the earlier Cochrane review, Paci et al. [18] concluded that screening lead to a reduction to mastectomy rates. Zorzi et al. [25] concluded that screening did not increase mastectomy rates.

A strength of this study is the use of detailed information from the cantonal cancer registry. The cancer registry collected detailed information on the tumours including the reason for cancer detection and staging information. A limitation of the study is that we had only the TNM stage available. In a follow-up study it is necessary to gather data on further possible influencing factors and analyse their influence on mastectomy rates. We are especially interested in the type of treatment provider, such as breast centres, medical reasons for type of surgery and distance to radiotherapy institutions. In that setting also potential differences in treatment of in situ cancers can be investigated. Since for these cancers, the treatment decision for mastectomy is most likely even more driven by medical reasons.

## Conclusion

Our study showed MSPs have a positive effect on lowering mastectomy rates. Screening participants are significantly less likely to receive a mastectomy compared to non-participants, which must be attributed to additional factors than just lower stages.

## Data Availability

Data is made available from the corresponding author upon reasonable request.

## Abbreviations

BC: Breast cancer
BCS: Breast-conserving surgery
DCO: Available case information due to death-certificate-only
MSP: Organized Mammography Screening Programme
Non-MSP: Outside of MSP
OR: Odds ratio
